# Systematic review of time trends in the prevalence of *Helicobacter pylori* infection in China and the USA

**DOI:** 10.1186/s13099-016-0091-7

**Published:** 2016-03-15

**Authors:** Peter Nagy, Saga Johansson, Michael Molloy-Bland

**Affiliations:** AstraZeneca Gothenburg, Pepparedsleden 1, 431 83 Mölndal, Sweden; School of Medicine, Pharmacy and Health, Durham University, Durham, UK; Research Evaluation Unit, Oxford PharmaGenesis Ltd, Oxford, UK

**Keywords:** *Helicobacter pylori*, Prevalence, China, USA, Time trends

## Abstract

It has been suggested that the prevalence of *Helicobacter pylori* infection has stabilized in the USA and is decreasing in China. We conducted a systematic literature analysis to test this hypothesis. PubMed and Embase searches were conducted up to 19 January 2015. Trends in the prevalence of *H. pylori* infection over time were assessed by regression analysis using Microsoft Excel. Overall, 25 Chinese studies (contributing 28 datasets) and 11 US studies (contributing 11 datasets) were included. There was a significant decrease over time in the *H. pylori* infection prevalence for the Chinese studies overall (*p* = 0.00018) and when studies were limited to those that used serum immunoglobulin G (IgG) assays to detect *H. pylori* infection (*p* = 0.014; 20 datasets). The weighted mean prevalence of *H. pylori* infection was 66 % for rural Chinese populations and 47 % for urban Chinese populations. There was a significant trend towards a decreasing prevalence of *H. pylori* infection for studies that included only urban populations (*p* = 0.04; 9 datasets). This trend was no longer statistically significant when these studies were further restricted to those that used serum IgG assays to detect *H. pylori* infection, although this may have been because of low statistical power due to the small number of datasets available for this analysis (*p* = 0.28; 6 datasets). There were no significant trends in terms of changes in the prevalence of *H. pylori* infection over time for studies conducted in the USA. In conclusion, the prevalence of *H. pylori* infection is most likely decreasing in China, due to a combination of increasing urbanization, which we found to be associated with lower *H. pylori* infection rates, and possibly also decreasing rates of *H. pylori* infection within urban populations. This will probably result in a gradual decrease in peptic ulcer and gastric cancer rates in China over time.

**Electronic supplementary material:**

The online version of this article (doi:10.1186/s13099-016-0091-7) contains supplementary material, which is available to authorized users.

## Background

*Helicobacter pylori* infection is well recognized as the main pathogenic factor for peptic ulcer disease and chronic gastritis, and in a subset of patients it is a major risk factor for gastric cancer and mucosa-associated lymphoid tissue lymphoma [1–3]. *Helicobacter pylori* infection has also been associated with increased risks of colon cancer [4], idiopathic thrombocytopenic purpura, iron deficiency anemia, vitamin B_12_ deficiency, and, more recently, neurodegenerative disorders and metabolic syndrome [5]. Conversely, a reduced risk of developing gastroesophageal reflux disease (GERD) with reflux esophagitis has been linked to infection with *H. pylori* (particularly in East Asia) [6–8], although this association remains controversial due to some conflicting results [5, 9–11].

Given the potential health consequences of *H. pylori* infection it is important to understand its prevalence in a population at any given time. Perhaps even more importantly from a healthcare planning perspective, it is useful to look at past trends in the prevalence of *H. pylori* infection so that its future prevalence and impact may be estimated. Infection with *H. pylori* is believed to be acquired mainly during childhood, remaining for the lifetime of the individual unless eradicated. In developed countries, mother-to-child transmission is the dominant mechanism; horizontal transmission is less likely than in developing countries because of better sanitation [12]. In developing countries, horizontal transmission may play a concomitant role with intrafamilial infection, leading to a higher prevalence. Consistent with these hypotheses, the prevalence of *H. pylori* has been greatly reduced over time in parallel with the westernization of Asian cultures such as Japan [13]. It is likely that the prevalence of *H. pylori* infection in China is decreasing too owing to improvements in living standards associated with recent rapid industrialization and urbanization.

Gastric cancer was the most common cancer in the first quarter of the 20th century. Since then, the incidence has decreased rapidly in the USA but has remained high in the Far East [14]. Despite the obvious health implications of changes in the prevalence of *H. pylori* infection, studies reporting prevalence data in China have not, to our knowledge, been systematically reviewed. We therefore systematically reviewed studies reporting the prevalence of *H. pylori* infection in Chinese adults, with a focus on trends over time. For comparison, we also systematically reviewed studies reporting the prevalence of *H. pylori* infection in adults in the USA.

## Methods

### Systematic searches and study selection

**Fig. 1:**
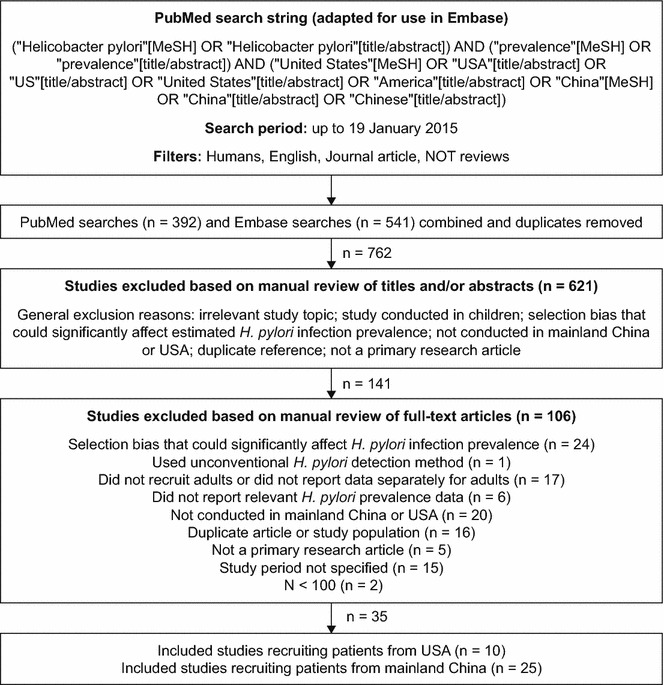
Preferred Reporting Items for Systematic Reviews and Meta-Analyses (PRISMA) flow diagram of the search strategy

Systematic searches were conducted in PubMed and Embase (via Ovid SP) up to 19 January 2015. Filters were applied to limit the searches to studies conducted in humans and published in English. Studies were considered for inclusion in the review if they reported the general prevalence of *H. pylori* infection in adults in mainland China or the USA. Exclusion criteria were: sample size less than 100 participants; absence of information on the time period during which the study was conducted; and population selection bias that could significantly impact on the reported prevalence of *H. pylori* infection (e.g., based on ethnicity, income, presence of upper gastrointestinal diseases [peptic ulcer, gastric cancer], or symptoms). Samples deemed acceptable for inclusion in terms of having minimal selection bias included those from population-based or health-check studies and healthy controls from case–control studies. Owing to the relatively low number of suitable US studies identified, samples from cohorts of patients with diseases not established (to our knowledge) as being associated with higher or lower *H. pylori* infection rates were also included, although data from healthy controls in such studies were preferentially selected if available. A Preferred Reporting Items for Systematic Reviews and Meta-Analyses [15] flow diagram of the search strategy is presented in Fig. 1.

### Data analysis

Prevalence data were plotted according to the midpoint of the study period (study midpoint). The study midpoint was defined as halfway (to the nearest 3 months) between the beginning and end of the reported study period. Trends in the prevalence of *H. pylori* infection over time were assessed by regression analysis using Microsoft Excel. In addition, because the trend analysis does not take into account the respective sample sizes of the contributing studies, Chinese and US studies were also grouped according to whether their study midpoint was before or after the mean of all study midpoints, and weighted mean prevalence estimates for *H. pylori* infection were calculated for these groups.

## Results

### Search results and study characteristics

Of 762 unique hits, 25 studies conducted in China [16–40] and 10 conducted in the USA [41–50] were included in the analysis. The study by Zhang et al. (2009) [31] included data from two separate rural populations (Muping County [Shandong province] and Yanqing County [Beijing Municipality]) and thus contributed two separate datasets for the purpose of the analysis. The studies by Chen et al. (2003) [20] and Chen et al. (2007) [22] each included data at two distinct time points from a single urban population (Shanghai and Guangzhou city, respectively), and thus also contributed two datasets each to the analysis. Therefore, there were 28 datasets in the Chinese analysis overall, and 10 in the US analysis (i.e., one dataset from each US study).

Characteristics of the included datasets are presented in Table 1 for the Chinese studies and as supplementary material (Additional file 1: Table S1—online) for the US studies. The mean sample size was 1621 (range: 100‒8820) for the Chinese datasets and 1742 (range: 143‒7465) for the US datasets. The Chinese datasets came mainly (26/28 [93 %]) from general population or health-check studies, or from healthy controls in case–control studies; half (5/10) of the US datasets came from these sources. Serum immunoglobulin G (IgG) assays were used to detect *H. pylori* infection in all of the US datasets, and in all Chinese datasets with study midpoints before the mean of all study midpoints for that country. There were variations, however, in the *H. pylori* detection methods used in the Chinese datasets with study midpoints after the mean of all Chinese study midpoints. In addition, a substantially higher proportion of datasets from Chinese studies conducted before the mean of the study midpoints were from rural (rather than urban) populations (8/12), compared with the proportion from studies conducted afterwards (3/16). Finally, although all included data were from adults, age distributions varied across both the Chinese and US datasets, as did the statistical measures used to report age characteristics. Datasets from US studies with a midpoint that was after the mean of all US study midpoints tended to include older patients than those from studies with a midpoint that was before the mean of the study midpoints. There were no clear differences in the age distributions of individuals in datasets from Chinese studies with study midpoints before versus after the mean of all study midpoints.

**Table 1:** Characteristics of selected studies reporting the prevalence of *Helicobacter pylori* infection in mainland China

Reference	Location	Population	N	Age of study population, years	Women, %	Rural/urban	Test	Study midpoint	*H.* *pylori* infection, %
Studies with midpoints *before* the mean of all study midpoints									
Forman et al. 1990 [16]	46 rural counties	General	1882	35‒64	50	Rural	Serum IgG	1983.75	60.4
			595	35–39					55.8
			913	40–49					56.6
			1061	50–59					65.8
			455	60–64					62.5
Yuan et al. 1999 [17]	Shanghai city	Healthy controls (cases had gastric cancer)	548	45‒64	NS	Urban	Serum IgG	1988	82
Zhang et al. 1996 [18]	14 randomly selected rural villages in Linqu County (Shandong province)	General (endoscopic survey)	2646	35‒64	47	Rural	Serum IgG	1989	72
Wang et al. 2008 [19]	67 rural counties	General	4020	35‒64; distributed equally across 10-year age groups	NS	Rural	Serum IgG	1989	71.4
Chen et al. 2003 [20]	Shanghai	Health check	668	≥20	38	Urban	Serum IgG	1990	44.5
			116	20–29					41.4
			132	30–39					46.2
			112	40–49					47.3
			104	50–59					50.0
			204	≥60					40.7
Chen et al. 2002 [21]	Shanghai	Health check	822	≥20	NS	Rural	Serum IgG	1990	62.0
			147	20–29					58.5
			182	30–39					64.3
			168	40–49					65.5
			121	50–59					68.6
			204	≥60					55.9
Chen et al. 2007 [22]	Guangzhou city	Health check	456	≥20	48	Urban	Serum IgG	1993	70.2
			135	20–29					65.2
			102	30–39					72.6
			109	40–49					76.2
You et al. 1998 [23]	Village in Cangshan County (Shandong province), which has a low incidence of gastric cancer	General	197	35‒64	50	Rural	Serum IgG	1994	55
Ma et al. 1998 [24]	Single village in Linqu County (Shandong province), which has a high incidence of gastric cancer	General (endoscopic survey)	289	35‒64	48	Rural	Serum IgG	1994	67.5
			131	35–44					77.3
			81	45–54					63.0
			77	55–64					62.3
Wong et al. 1999 [25]	Seven villages (Wuhong, Yingchen, Meihua, Xuojan, Yutien, Jiangtien, and Hunane) in Changle County, which has a high incidence of gastric cancer	Health check	1828	18–76 Mean: 43.0	NS	Rural	Serum IgG	1994.5	80.9
Cai et al. 2000 [26]	Changle County (Fujian province), which has a high incidence of gastric cancer	Healthy controls (cases had gastric cancer)	100	≥30	14	NS	Serum IgG	1997.25	68
			5	30–39					–
			18	40–49					–
			22	50–59					–
			38	60–69					–
			18	≥70					–
Brown et al. 2001 [27]	Linqu County (Shandong province)	General	3228	NS	63	Rural	Serum IgG	1998	60.6
			770	35–39					63.0
			848	40–44					62.5
			893	45–54					60.0
			299	55–59					57.0
			478	≥60					57.0
Studies with midpoints *after* the mean of all study midpoints									
Chen et al. 2003 [20]	Shanghai	Health check	1272	≥20	37	Urban	Serum IgG	2001	65.4
			168	20–29					52.4
			251	30–39					60.6
			460	40–49					70.0
			151	50–59					72.8
			242	≥60					66.1
Chen et al. 2007 [22]	Guangzhou city	Health check	1001	≥20	NS	Urban	Serum IgG	2003.5	56.4
			253	20–29					53.4
			196	30–39					54.6
			204	40–49					63.2
			163	50–59					57.7
Cheng et al. 2009 [28]	Beijing	Health check	1161	≥20	47	Both	Breath test	2003.75	46.6
			46	20–29					41.3
			222	30–39					49.5
			397	40–49					45.6
			369	50–59					44.4
			94	≥60					53.2
Chen et al. 2008 [29]	Five regions of Zhejiang province	Cirrhotic patients	457	Mean: 57.3 SD: 13	26	NS	Biopsies (rapid urease test and histology)	2004.25	60.6
Shi et al. 2008 [30]	Three rural villages in Xiangshui and Gaoyou (Northern Jiangsu province)	General	1322	≥20	57	Rural	Serum IgG and breath test	2005	62.1
			48	20–30					56
			84	30–40					67.3
			287	40–50					57.6
			297	50–60					64.3
			372	>60					60.6
Zhang et al. 2009 [31]	Yanqing County (Beijing municipality), which has a low incidence of gastric cancer	General	503	40‒79	53	Rural	Stool antigen assay	2006.5	41.4
			178	40–49					37.1
			215	50–59					44.2
			110	60–79					42.7
Zhang et al. 2009 [31]	Muping County (Shandong province), which has a high incidence of gastric cancer	General	526	40‒79	53	Rural	Stool antigen assay	2006.5	51
			214	40–49					47.7
			208	50–59					53.4
			104	60–79					52.9
Peng et al. 2009 [32]	Guangzhou city	Health check	2580	≥18 Mean: 45.1 SD: 14.2	50	Urban	Breath test and biopsy	2006.75	28
Li et al. 2010 [33]	Five regions in Shanghai	General	3151	18–80 Mean: 47.7 SD: 14.1	56	Both	Serum IgG	2007.75	73.3
Wang et al. 2011 [34]	Shandong province	Health check	1637	Mean: 47	39	NS	Serum IgG	2008.75	35.5
Xia et al. 2012 [35]	Shijiazhuang city	Migrant workers	324	Mean: 37.8	59	Urban	Breath test	2009.5	41.0
			211	<40					44.1
			113	≥40					35.4
Wang et al. 2012 [36]	Xi’an, north-west China, which has a high incidence of gastric cancer	Healthy controls (cases had gastric cancer)	514	Mean: 58.2	27	NS	Serum IgG	2009.5	35
Jing Jiang et al. 2012 [37]	Jilin province	Healthy controls from routine health check (cases were patients with gastric cancer)	932	35–80	42	Both	Serum IgG	2009.5	49.7
Hu et al. 2012 [38]	Dalian Port	Health check (maritime workers)	3995	≥18	21	Urban	Serum IgG	2010.5	44.9
			945	18–29					43.1
			1245	30–39					41.2
			1111	40–49					50.1
Cao et al. 2014 [39]	Tianjin	Healthy controls (cases had fundic gland polyps)	530	NS	NS	Urban	Breath test, rapid urease test, or histology	2011.75	42.3
Xu et al. 2014 [40]	Zhejiang province	Health check	8820	Median: 46.0 Range: 39‒53	40	NS	Breath test	2013	43.8

*IgG* immunoglobulin G, *NS* not specified, *SD* standard deviation

### *H. pylori* infection prevalence over time in China

**Fig. 2:**
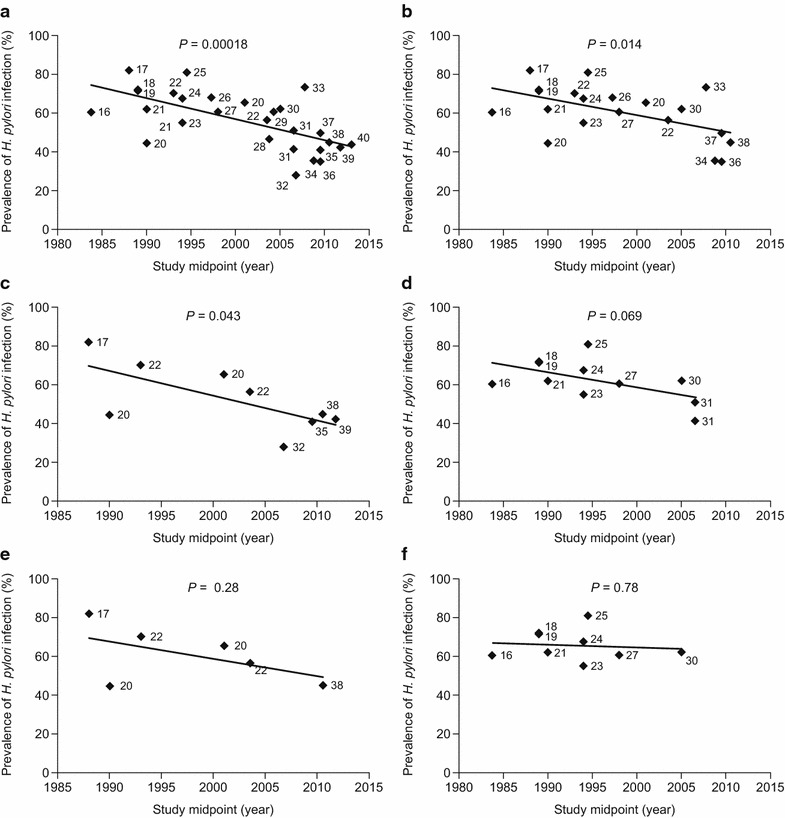
Prevalence estimates of *Helicobacter pylori* infection over time in Chinese studies (numbers by data points indicate source reference citations). Data are reported for **a** all included datasets (n = 28), **b** datasets that used serum IgG assays to detect *H. pylori* infection (n = 20), **c** datasets that included individuals recruited from urban areas only (n = 9), **d** datasets that included individuals recruited from rural areas only (n = 11), **e** datasets that used serum IgG assays to detect *H. pylori* infection and included individuals recruited from urban areas only (n = 6), and **f** datasets that used serum IgG assays to detect *H. pylori* infection and included individuals recruited from rural areas only (n = 9). *IgG* immunoglobulin G

**Fig. 3:**
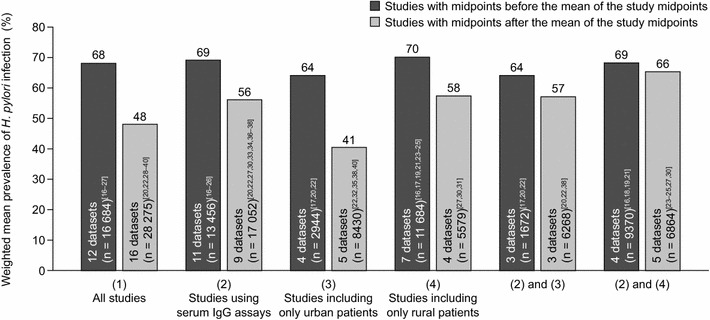
Mean prevalence estimates (weighted by sample size) of *Helicobacter pylori* infection for Chinese datasets with study midpoints before, and after, the mean of all study midpoints, according to the assay method used and whether or not patients were living in urban or rural areas. *IgG* immunoglobulin G

The weighted mean prevalence of *H. pylori* infection across all years was 55 % (range: 28‒82 % [1983‒2013]) for the Chinese datasets (Table 1). There was a significant decrease over time in the prevalence of *H. pylori* infection when all the Chinese datasets were included in the analysis (*p* = 0.00018; Fig. 2a). Consistent with this trend, the weighted mean prevalence of *H. pylori* infection was higher for Chinese datasets with study midpoints that fell before than for those with study midpoints that fell after the mean of all study midpoints (68 vs 48 %; Fig. 3).

The trend of decreasing prevalence of *H. pylori* infection over time remained when the analysis was limited to datasets that used serum IgG assays to detect *H. pylori* infection (*p* = 0.014; Fig. 2b). Similarly, the weighted mean prevalence of *H. pylori* infection was higher for datasets with midpoints that fell before, versus after, the mean of all study midpoints (69 vs 56 %; Fig. 3).

The weighted mean prevalence of *H. pylori* infection was higher for datasets that included only rural populations (66 %; range: 41‒81 % [1983‒2007]) than for those that included only urban populations (47 %; range: 28‒82 % [1988‒2012]). With regard to trends over time, similar patterns of decreasing prevalence of *H. pylori* infection were observed when datasets were grouped according to whether they included only urban or rural populations (Fig. 2c, d, and Fig. 3), although the trend test was only significant for the urban analysis (*p* = 0.04 [urban]; *p* = 0.07 [rural]). The strength of the trends, however, decreased (especially for the rural analysis) and were not statistically significant (*p* = 0.28 [urban]; *p* = 0.78 [rural]) when only datasets that used serum IgG assays to detect *H. pylori* infection were included (Fig. 2e, f, and Fig. 3), although this may have been because of reduced statistical power associated with the smaller available sample size, particularly for the urban serum IgG analysis.

**Fig. 4:**
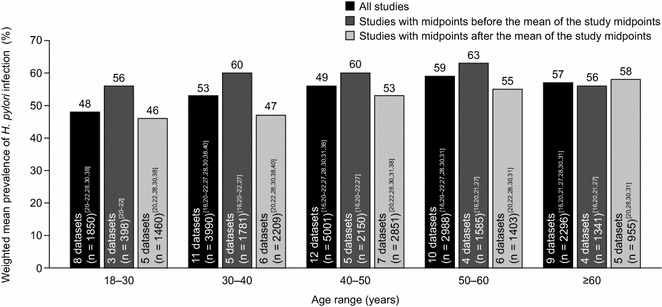
Mean prevalence estimates (weighted by sample size) of *Helicobacter pylori* infection for Chinese datasets overall and with study midpoints before, and after, the mean of all study midpoints, according to age group

The weighted mean prevalence of *H. pylori* infection across all datasets reported by age group was lowest among individuals aged 18–30 years (48 %; range: 43–65 % [1990–2011]) and increased with age up to the 50–60-year age category (59 %; range: 44–73 % [1984–2007]) (Fig. 4). The weighted mean prevalence of *H. pylori* infection was higher for datasets with midpoints that fell before the mean of all study midpoints than for those with midpoints that fell after the mean of all study midpoints in every age group except patients aged 60 years and over (Fig. 4). No significant trends over time, however, were detected by regression analysis when prevalence data for each age group were plotted according to the study midpoint (data not shown).

### *H. pylori* infection prevalence over time in the USA

The weighted mean prevalence of *H. pylori* infection across all years was 35 % (range: 22–48 % [1990–2006]) for the US datasets (Additional file 1: Table S1—online). There was no significant trend in terms of changes in the prevalence of *H. pylori* infection over time for the US datasets (*p* = 0.34). The weighted mean prevalence of *H. pylori* infection was lower for US datasets with midpoints that fell before the mean of all study midpoints than for those with midpoints that fell after the mean of all study midpoints (32 [41–45] vs 40 % [46–50]). Of the five datasets from studies with midpoints that fell before the mean of all study midpoints, however, only two included patients over 40 years of age [42, 45], compared with all five datasets from studies with midpoints that fell after the mean of all study midpoints. Furthermore, three of the five datasets that had a study midpoint after the mean of all study midpoints did not specify the ethnic makeup of the populations assessed [46, 48, 49].

To explore the impact of age and ethnicity on *H. pylori* infection further, we extracted prevalence data reported for specific ethnicities and age groups. For these analyses, the sample size exclusion criterion was not applied, hence the inclusion of one additional study by Castillo et al. 2004 [51] for the ethnicity analysis, which was conducted in 33 healthy controls (cases had dyspepsia) from Olmstead County, MN (mean age: 61 years; 58 % women; 100 % white).

**Fig. 5:**
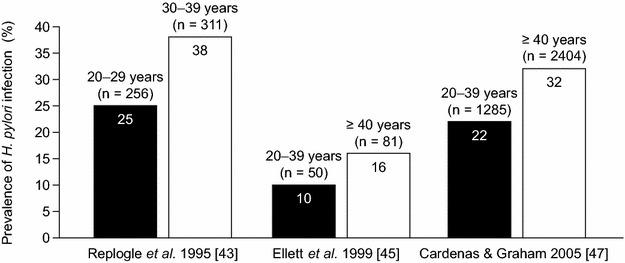
Mean prevalence estimates (weighted by sample size) of *Helicobacter pylori* infection in studies conducted in the USA, according to age group

Three studies conducted in the USA provided data on the prevalence of *H. pylori* infection according to different age groups [43, 45, 47]. In all of these studies, the prevalence of *H. pylori* infection was higher in older than in younger individuals (Fig. 5). There were insufficient data available to assess trends in *H. pylori* infection over time for different age groups.

**Fig. 6:**
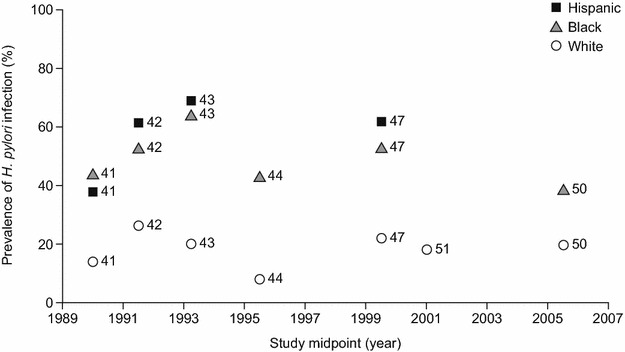
Prevalence estimates of *Helicobacter pylori* infection over time in US studies, according to ethnicity (numbers by data points indicate source reference citations). Note: sample size was not reported for reference 44 and data from this study are therefore not included in weighted mean prevalences

Seven studies conducted in the USA provided data on the prevalence of *H. pylori* infection according to ethnicity [41–44, 47, 50, 51], although one of these did not provide the sample size [44] and was thus not included in weighted mean prevalence calculations. The prevalence of *H. pylori* infection was consistently higher for Hispanic and black individuals than for white individuals, and the prevalence of *H. pylori* within the different ethnicities was fairly consistent in terms of trends over time (Fig. 6), particularly for the weighted mean prevalences before and after the mean of all study midpoints, which were nearly identical (Hispanic: 61 vs 62 %; black: 52 vs 52 %; white: 24 vs 22 %). It should be noted that the weighted mean prevalence data were driven mainly by two large studies (Cardenas et al. 2005 [47] and Kruszon-Moran et al. 2005 [42]), which were both of high quality and used data during different time periods (1988‒1994 and 1999‒2000, respectively) from the same source, the National Health and Nutritional Examination Survey.

## Discussion

Understanding country-specific trends in the prevalence of *H. pylori* is an essential component of successful future healthcare planning. We conducted a systematic literature analysis of trends in *H. pylori* infection over time in China, where rapid industrialization is probably altering the prevalence of this important organism, and the USA, where the prevalence may have stabilized. Despite variation in the characteristics of the included studies, a significant decrease in the prevalence of *H. pylori* infection over time was observed for datasets from studies conducted in China, which was consistent with a higher weighted mean prevalence of *H. pylori* infection for datasets from studies with a midpoint before, rather than after, the mean of all the study midpoints.

It was noted that there was greater variation in methods used to detect *H. pylori* infection in datasets from more recent studies conducted in China than for older studies. Given that detection methods for *H. pylori* infection vary in terms of their sensitivity and specificity, and positive and negative predictive values [52], this factor could influence the observed trend in prevalence estimates over time. The trend was still significant, however, when only datasets from studies that used serum IgG assays (the most common method used to detect *H. pylori*) were included.

In addition, a higher proportion of the datasets from more recent Chinese studies were from urban rather than rural populations than for older studies. Improved living standards associated with urbanization may reduce the prevalence of *H. pylori* infection, which could influence the observed trend. Indeed, the overall weighted mean prevalence of *H. pylori* infection was much higher in samples that included individuals from rural areas only than in those that included individuals from urban areas only in our study (66 vs 47 %). However, there was also a significant trend towards a decreasing prevalence of *H. pylori* infection reported over time in studies that included only urban populations. The statistical significance of this association was lost when the analysis was further restricted to studies that used serum IgG assays, although the direction of the trend was still apparent across the few remaining datasets (n = 6) available for this analysis. This suggests either that the decreasing trend in *H. pylori* prevalence reported in Chinese urban studies over time is an artifact caused by an increase in those using non-serological methods to detect *H. pylori* infection, or that the significance of the trend was merely obscured owing to the reduction in sample size. The latter seems more plausible, since in the broader analysis the inclusion of only studies using serum IgG assays did not substantially visually affect the strength of the decreasing trend over time (the statistical significance decreased from *p* = 0.00018 to *p* = 0.014, but this might be expected given that the sample size was reduced from 28 to 20). This is consistent with recent evidence showing that differences in the sensitivity and specificity of different *H. pylori* detection methods are not that great. It is therefore questionable whether such differences would have a major impact on the observed trends in prevalence estimates over time [53].

Our study shows that urban populations in China have much lower rates of *H. pylori* infection than rural populations. The inevitable conclusion, since there has been rapid urbanization in China (possibly explaining the increase in studies conducted in urban areas over time, although this may be coincidental), is that the general prevalence of *H.pylori* infection must also have decreased. In addition, the prevalence of *H. pylori* infection may have decreased over time within urban populations. This is entirely plausible, since mean household incomes in urban areas of China have increased nearly seven-fold from 4272 yuan to 29,547 yuan since 1995 (information sourced from http://www.statista.com) and this would be expected to improve living standards in ways that may reduce *H. pylori* infection, such as by lowering household crowding. Indeed, similar socioeconomic factors are thought to influence differences in the prevalence of *H. pylori* infection between ethnicities in the USA [54].

Interestingly, lower weighted mean prevalence estimates were observed for newer datasets than for older datasets for all age groups except patients aged 60 years and over. These observations are based on a small number of studies, but are consistent with a role for reduced horizontal transmission of *H. pylori* owing to improved sanitation associated with urbanization. Specifically, reduced mother-to-child transmission alone is unlikely to account for reductions in the prevalence of *H. pylori* in older individuals, because they would have been children before the onset of significant increases in urbanization in China.

As expected, the overall prevalence of *H. pylori* infection in the US datasets was lower than in the Chinese datasets, although the prevalence of *H. pylori* infection in datasets from Chinese studies with midpoints that fell after the mean of all study midpoints was lower (48 %) than that in datasets from the US studies of black (52 %) and Hispanic (62 %) individuals. There was no significant upward or downward trend in the prevalence of *H. pylori* infection over time in the USA, although the weighted mean prevalence of *H. pylori* infection was slightly lower for US datasets with midpoints that fell before the mean of the study midpoints than for those with midpoints that fell after the mean of the study midpoints. The latter observation was probably due in part to the inclusion of older populations in the more recent US studies. This is consistent with the finding that *H. pylori* infection was more common in older patients than in younger individuals in the only three US studies reporting such data.

US data on *H. pylori* infection were based on a smaller number of datasets than was available for the Chinese analysis and should thus be interpreted with caution. Nevertheless, the idea that *H. pylori* infection rates have stabilized in Hispanic, black, and white individuals in the USA is supported by two large population-based studies, which found virtually identical prevalence estimates over the span of a decade [42, 47]. Furthermore, these data are consistent with stabilized rates of infection observed in at least one European study [55]. The continued higher prevalence of *H. pylori* infection in black and Hispanic than in white individuals probably reflects sustained socioeconomic disparities in the USA.

In terms of healthcare consequences, decreasing rates of *H. pylori* infection in China are likely to lead to a gradual decrease in peptic ulcer and gastric cancer rates; the overall proportion of peptic ulcers related to other causes will consequently increase. Conversely, the prevalence of GERD and its complications may increase in China, if it is correct that *H. pylori* infection plays a role in the low prevalence of GERD in East Asian countries compared with the West [6, 56]. In the USA, the prevalence of peptic ulcer disease and gastric cancer may increase slightly over the coming decades, assuming that elevated *H. pylori* infection rates are maintained among US Hispanic populations, and that 2012 census projections (census.gov) for a doubling of the Hispanic population in the USA from 17.8 to 30.6 % prove to be correct.

## Conclusions

The prevalence of *H. pylori* infection appears to have decreased over time in China, while it has stabilized in the USA. Urbanization may reduce the prevalence of *H. pylori* infection.

## Additional file

10.1186/s13099-016-0091-7 Characteristics of selected studies reporting the prevalence of *Helicobacter pylori* infection in the USA.

## Abbreviations

IgG: immunoglobulin G
NS: not significant
PRISMA: Preferred Reporting Items for Systematic Reviews and Meta-Analyses
SD: standard deviation
