# Supplementary material for: Systematic review of time trends in the prevalence of Helicobacter pylori infection in China and the USA
Source: Gut Pathog. 2016 Mar 15;8:8. doi: 10.1186/s13099-016-0091-7 (PMC4791971; doi:10.1186/s13099-016-0091-7)
Supplement: Supplementary file 1 — 10.1186/s13099-016-0091-7 Characteristics of selected studies reporting the prevalence of Helicobacter pylori infection in the USA. [file 13099_2016_91_MOESM1_ESM.docx]

**Table S1** **Characteristics of selected studies reporting the prevalence of *Helicobacter pylori* infection in the USA**

| **Reference** | **Location** | **Population** | **N** | **Age of study population, years** | **Women, %** | **Ethnicity** | **Test** | **Study midpoint** | ***H. pylori* infection, %** |
| --- | --- | --- | --- | --- | --- | --- | --- | --- | --- |
| Studies with midpoints *before* the mean of all study midpoints | | | | | | | | | |
| Smoak  *et al*. 1994 [41] | Fort Jackson, SC | New army recruits | 938 | 17‒26 | 43 | Overall | Serum IgG | 1990 | 26.3 |
|  |  |  | 536 |  |  | White |  |  | 14 |
|  |  |  | 324 |  |  | Black |  |  | 44 |
|  |  |  | 47 |  |  | Hispanic |  |  | 38 |
| Kruszon-Moran  *et al*. 2005 [42] | National | General (National Health and Nutritional Examination Survey [NHANES]) | 7465 | ≥20 | 50 | Overall | Serum IgG | 1991.5 | 32.7 |
|  |  |  | 3353 |  |  | White |  |  | 26.2 |
|  |  |  | 1851 |  |  | Black |  |  | 52.7 |
|  |  |  | 2021 |  |  | Hispanic |  |  | 61.6 |
| Replogle  *et al*. 1995 [43] | Northern CA | Health check | 567 | 20‒24 (n = 114)  25‒29 (n = 142)  30‒34 (n = 156)  35‒39 (n = 155) | 53 | Overall | Serum IgG | 1993.25 | 27.2 |
|  |  |  | 201 |  |  | White |  |  | 20 |
|  |  |  | 198 |  |  | Black |  |  | 64 |
|  |  |  | 157 |  |  | Hispanic |  |  | 69 |
| Malaty  *et al*. 2002 [44] | Bogalusa, LA | General | 143 | 18‒23 | 51 | Overall | Serum IgG | 1995.5 | 24.5 |
|  |  |  | NS |  |  | White |  |  | 8 |
|  |  |  | NS |  |  | Black |  |  | 43 |
| Ellett *et al*. 1999 [45] | Indianapolis and Valparaiso, IN | Blood donors | 250 | 20‒29 (n = 7)  30‒39 (n = 43)  40‒49 (n = 59)  ≥50 (n = 22)  Mean: 38.6 SD: 10.1 | 100 | NS | Serum IgG | 1996 | 17.9 |
| Studies with midpoints *after* the mean of all study midpoints | | | | | | | | | |
| Bunch  *et al*. 2008 [46] | Salt Lake City, UT | Patients with suspected coronary artery disease who did not have atrial fibrillation on investigation | 660 | Mean: 63.9 SD: 10.7 | 23 | NS | Serum IgG | 1998 | 55 |
| Cardenas  *et al*. 2005 [47] | National | General (National Health and Nutritional Examination Survey [NHANES]) | 3689 | 20‒29 (n = 644)  30‒39 (n = 641)  40‒49 (n = 602)  50‒59 (n = 475)  ≥60 (n = 1327) | 53 | Overall | Serum IgG | 1999.5 | 32 |
|  |  |  | 1713 |  |  | White |  |  | 22 |
|  |  |  | 634 |  |  | Black |  |  | 53 |
|  |  |  | 1228 |  |  | Hispanic |  |  | 61.9 |
| Lutsey  *et al*. 2009 [48] | Six US communities | Patients free from cardiovascular disease (MultiEthnic Study of Atherosclerosis [MESA]) | 1000 | 45‒84 | NS | NS | Serum IgG | 2001.5 | 45.4 |
| Erim *et al*. 2008 [49] | Cleveland, OH | Healthy controls (cases were morbidly obese patients undergoing  Roux-en-Y gastric bypass) | 2444 | ≥15 | NS | NS | Serum IgG | 2004.5 | 48.2 |
| Papasavas *et al*. 2008 [50] | Pittsburgh, PA | Patients undergoing laparoscopic Roux-en-Y gastric bypass | 259 | Mean: 44.9 Range: 18‒69 | 82 | Overall | Serum IgG | 2005.5 | 22.4 |
|  |  |  | 219 |  |  | White |  |  | 19.6 |
|  |  |  | 39 |  |  | Black |  |  | 38.5 |

IgG, immunoglobulin G; NS, not specified; SD, standard deviation.
